# LRRK2 Contributes to Secondary Brain Injury Through a p38/Drosha Signaling Pathway After Traumatic Brain Injury in Rats

**DOI:** 10.3389/fncel.2018.00051

**Published:** 2018-03-01

**Authors:** Qin Rui, Haibo Ni, Fan Gao, Baoqi Dang, Di Li, Rong Gao, Gang Chen

**Affiliations:** ^1^Department of Laboratory, The First People’s Hospital of Zhangjiagang, Suzhou, China; ^2^Department of Neurosurgery, The First People’s Hospital of Zhangjiagang, Suzhou, China; ^3^Department of Rehabilitation, Zhangjiagang Hospital of Traditional Chinese Medicine Affiliated to Nanjing University of Chinese Medicine, Suzhou, China; ^4^Department of Translational Medicine Center, The First People’s Hospital of Zhangjiagang, Suzhou, China; ^5^Department of Neurosurgery and Brain and Nerve Research Laboratory, The First Affiliated Hospital of Soochow University, Suzhou, China

**Keywords:** LRRK2, p38, Drosha, traumatic brain injury, secondary brain injury

## Abstract

Leucine-rich repeat kinase 2 (LRRK2) is widely expressed in the brain and exerts neurotoxicity in Parkinson’s disease. The p38/Drosha signaling activation has been reported to increase cell death under stress. This study was designed to investigate the potential role and mechanism of LRRK2 in secondary brain injury after traumatic brain injury (TBI). A total of 130 male Sprague-Dawley rats were examined using a weight-drop model of TBI. The rats received the specific LRRK2 inhibitor PF-06447475 or LRRK2 pDNA alone or in combination with Drosha pDNA. Real-time PCR, western blot, immunofluorescence, neuronal apoptosis, brain water content, and neurological score analyses were conducted. Our results showed that after TBI, endogenous LRRK2 expression and p38 phosphorylation were increased, whereas Drosha expression was inhibited. Administration of the LRRK2 inhibitor PF-06447475 significantly reduced neuronal apoptosis, brain water content, and blood–brain barrier permeability 12 h after TBI and ameliorated neurological deficits 72 h after TBI, which was concomitant with decreased p38 phosphorylation and increased Drosha expression. Conversely, LRRK2 overexpression induced the opposite effect. Moreover, the neurotoxic effects of LRRK2 on TBI were also eliminated by Drosha overexpression. Altogether, these findings demonstrate the importance of TBI-induced LRRK2 upregulation during the induction of post-traumatic neurological injury, which may be partially mediated through a p38/Drosha signaling pathway.

## Introduction

Traumatic brain injury (TBI) is a major cause of morbidity and mortality among comparatively young individuals worldwide, and it results in serious declines in quality of life (Dash et al., 2016; Zhao et al., 2017; Dixon, 2017). TBI-induced harmful cerebral effects involve a combination of primary and secondary damage (Dixon, 2017). Primary brain injury following TBI is untreatable; however, due to glutamate toxicity, oxidative stress, and inflammation, the delayed and prolonged secondary brain injury, which results in increased blood–brain barrier (BBB) permeability, cerebral edema, and neurological impairment, can largely determine the prognosis and outcome of TBI patients (Laird et al., 2014; Dixon, 2017). Therefore, extensive research efforts regarding the mechanisms underlying the pathogenesis of secondary brain injury after TBI are urgently needed.

Leucine-rich repeat kinase 2 (LRRK2), the most commonly mutated gene in both familial and sporadic Parkinson’s disease (PD), is a large (2527 amino acids, 286 kDa) multidomain protein and is widely expressed in many tissues and cells, especially in the brain (Islam and Moore, 2017; Kang and Marto, 2017). The high kinase activity of LRRK2 is associated with defects in protein synthesis and degradation, apoptosis, inflammatory responses, and oxidative damage (Tsika and Moore, 2012; Cookson, 2015; Islam and Moore, 2017), which are also factors that trigger acute brain injury following TBI (Devyatov et al., 2017; Dixon, 2017). In addition to its toxic effects in PD, LRRK2 also promotes post-ischemic apoptotic cell death by modulating Tau phosphorylation in experimental cerebral ischemia (Kim and Vemuganti, 2017). Specifically, LRRK2 can activate MAPK signaling cascades, such as the p38 MAPK pathway, by phosphorylating MAPK kinases (MKKs) (Gloeckner et al., 2009; Hsu et al., 2010a). Under stress conditions, activated p38 inhibits the cellular survival program that is mediated by Drosha, which is a functional nuclease that processes long primary microRNAs (pri-miRNAs) into precursor microRNAs (pre-miRNAs) with its cofactor in the nucleus (Hong et al., 2013; Yang et al., 2015; Mechtler et al., 2017). After TBI, phosphorylated p38 (p-p38) is significantly increased in the brain and participates in the pathological processes of TBI (Tang et al., 2012; Yang et al., 2017). However, until now, no study has addressed whether or how LRRK2 is involved in TBI.

Therefore, in this study, we aimed to characterize the role and mechanisms of LRRK2 using a weight-drop TBI rat model. We hypothesized that (1) endogenous LRRK2 expression is stimulated and plays a key role in inducing secondary brain injury following TBI and that (2) the toxic effects by LRRK2 are partially mediated through a p38/Drosha signaling pathway.

## Materials and Methods

### Animals

One hundred and thirty male Sprague-Dawley rats (weighing 250–280 g) were purchased from the Animal Center of the Chinese Academy of Sciences (Shanghai, China). Animals were housed in 12-h light/dark cycles at a controlled temperature and humidity with free access to food and water. All experimental protocols were approved by the Animal Care and Use Committee of Soochow University and were implemented according to the Animal Research: Reporting of In Vivo Experiments (ARRIVE) guidelines.

### TBI Model

The TBI model was generated based on a previously described protocol (Hang et al., 2004). Briefly, animals were fixed in the stereotactic instrument after intraperitoneal anesthetization with 4% chloral hydrate (400 mg/kg body weight). Behind the cranial coronal suture and beside the midline, we made a right parietal bone window of 5 mm in diameter with a bone drill under aseptic conditions, and the skull disk was removed without disturbing the dura. Only animals with intact dura were used for inducing contusion model. A copper weight (4 mm in diameter, 5 mm in height) was placed onto the cranial dura, and trauma was introduced by setting up a 40-g, flat-end steel rod and inducing its fall onto the copper weight from a height of 25 cm. The pillar was allowed to compress the brain tissue to a maximum depth of 3 mm. The blood was carefully wiped away with gauze after the weight was removed, and the scalp was sutured with a silk thread. The animals were allowed to recover in a warmed chamber before being returned to their home cages. The sham group animals underwent the exact same procedure but were not impacted with the steel rod.

### Tissue Collection and Sectioning

**FIGURE 1:**
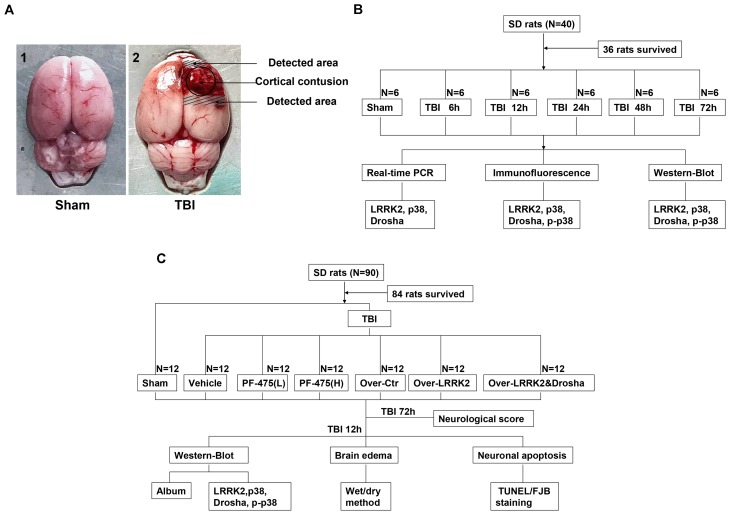
Experimental design. **(A)** Brain tissue from the peripheral injury (peri-injury) in the TBI group and from the same location in the sham group was obtained for the assay. **(B)** Experiment I was designed to demonstrate the expression levels and locations of LRRK2/p38/Drosha over time after TBI and determine a suitable time point for the second experiment. **(C)** Experiment II was designed to observe the effects of LRRK2 on early brain injury after TBI and explore the potential mechanisms.

Rats were deeply anesthetized with sodium pentobarbital (100 mg/kg, intraperitoneal injection) at an equivalent time point after injury. For the isolation of proteins and messenger RNAs (mRNAs), rats were transcardially perfused with 200 ml of 4°C 0.9% saline, and a sample of the cortex surrounding the contusion area that was located <3 mm from the margin of the contusion site (or the region located <3 mm from the parietal craniotomy in the sham group) was collected on ice (**Figure 1A**). The obtained tissue samples were rapidly frozen in liquid nitrogen and stored at -80°C until further use.

For brain sections, rats were transcardially perfused with 200 ml of 4°C 0.9% saline, followed by 250 ml of ice-cold 4% paraformaldehyde (pH 7.4). Brains were removed, immersed overnight in 4% paraformaldehyde at 4°C, and cryoprotected in a 15% sucrose solution overnight, followed by a 1-day incubation in a 30% sucrose solution. Frozen brain sections were cut at a 15-μm thickness using a sliding microtome (Leica CM1950, Germany). Serial coronal sections were collected between -2.12 mm bregma and -4.80 mm bregma. Every fifth section initiated from a random start point was selected for the appropriate staining procedure. All the processes used for tissue resection and selection were conducted by two pathologists who were blinded to the experimental conditions.

### Study Design and Experimental Groups

Two separate experiments were conducted (**Figures 1B,C**).

#### Experiment I

To perform a time-course analysis of endogenous LRRK2, p38, and Drosha after TBI, 36 rats (40 rats were used and 36 rats survived after the surgery) were randomly divided into the sham group and 5 experimental groups arranged by time as follows: 2, 12, 24, 48, and 72 h after TBI. The relative expression levels of the target molecules were measured by real-time PCR and western blotting. Additionally, double immunofluorescence analysis was performed to characterize the cellular localization of LRRK2, p38, and Drosha at 24 h after TBI (**Figure 1B**).

#### Experiment II

In the second experiment, 84 rats (90 rats were used and 84 rats survived) were randomly divided into the following seven groups: the sham group, TBI+DMSO vehicle group (Vehicle, intraperitoneal injection), TBI+low-dose PF-06447475 group [PF-475(L), 3 mg/kg, IP], TBI+high-dose PF-06447475 group [PF-475(H), 5 mg/kg, intraperitoneal injection], TBI+over-vector group (Over-Ctr, intracerebroventricular injection, ICV), TBI+LRRK2 overexpression group (Over-LRRK2, ICV), and TBI+LRRK2 overexpression+Drosha overexpression group (Over-LRRK2&Drosha, ICV). At 12 h after TBI, which was the time point determined from the results of experiment I, peripheral injury (peri-injury) brain cortices were extracted for western blot, brain water content, terminal deoxynucleotidyl transferase-mediated dUTP nick-end labeling (TUNEL) staining and Fluoro-Jade B (FJB) staining. Moreover, neurological testing was performed at 72 h after TBI (**Figure 1C**).

### Drug Administration

Intracerebroventricular injection drug administration was performed as previously described (Shen et al., 2015). Briefly, rats were placed in a stereotaxic apparatus under 4% chloral hydrate anesthesia, and the drugs were inserted using a 100-μl Hamilton syringe (Hamilton Company, United States) through a burr hole into the right lateral ventricles at the following coordinates relative to the bregma: 1.5 mm posterior, 1.0 mm lateral, and 3.2 mm below the horizontal plane of the skull (Shen et al., 2015). Specific pDNA for LRRK2 and Drosha was obtained from Guangzhou Ribo Biotechnology Co., Ltd. (Guangzhou, China). Full-length LRRK2 or Drosha cDNA was cloned into pcDNA3.1(+) using the cytomegalovirus promoter. The pDNA was dissolved in the transfection reagent and diluted with 0.9% saline. Twenty microliters of pDNA (500 pmol/10 μl) or vehicle (transfection reagent in saline) was administered by ICV injection at a rate of 0.5 μl/min at 24 h before TBI modeling. The cannula remained in place for an additional 5 min and was then withdrawn slowly over 5 min. Finally, the incision was closed with sutures, and the rats were allowed to recover. Additionally, LRRK2 inhibitor PF-475 (Sigma, United states) was dissolved in dimethyl sulfoxide (DMSO) and diluted with 0.9% saline to a final concentration of <1% DMSO. One milliliter of PF-475 (3, 5 mg/kg) or vehicle (DMSO in saline) was injected intraperitoneally at 1 h post-TBI modeling (Atashrazm and Dzamko, 2016).

### Real-Time PCR

Total RNA was isolated from peri-injury brain tissues using the TRIzol Reagent (Invitrogen, United States) according to the manufacturer’s instructions. According to the protocol provided by the manufacturer (Thermo Fisher, United States), complementary DNA (cDNA) was synthesized using 1 μg of the total RNA. Real-time PCR was then performed using the QuantStudio^TM^ Dx Real-Time PCR Instrument (Life Technologies Corporation, United States) with a PowerUp^TM^ SYBR^TM^ Green Master Mix kit (Thermo Fisher, United States). The phases included the following: the template was denatured at 95°C for 2 min, followed by 40 cycles of amplification (95°C for 15 s, 60°C for 15 s, and 72°C for 1 min). All the samples were analyzed in triplicate. The expression of glyceraldehyde 3-phosphate dehydrogenase (GAPDH) mRNA was used as the internal reference for each sample, and the relative mRNA expression levels of the target genes were calculated using relative quantification (2^-ΔΔCT^). The sequences of the forward and reverse primers of each gene are as follows:

LRRK2: F: 5′-AAAGGGCGACAACCAGGTCA-3′,
R: 5′-CCGGAGCACTTTCCTCGCTA-3′;
p38: F: 5′-TCGGCTGACATAATCCACAG-3′,
R: 5′-GTAGCCGGTCATTTCGTCAT-3′;
Drosha: F: 5′-CTCACCCTGACCGACTTCAT-3′,
R: 5′-GGATAAATGCTGTGCCGAAT-3′;
GAPDH: F: 5′-TGGCCTTCCGTGTTCCTACC-3′,
R: 5′-CGCCTGCTTCACCACCTTCT-3′.

### Western Blot Analysis

Western blot analysis was performed as previously described (You et al., 2016). Briefly, protein extraction from peri-injury cortex tissues was performed by gently homogenizing the samples in RIPA lysis buffer with phosphatase inhibitors (Beyotime, China) with further centrifugation at 13,000 × *g* at 4°C for 20 min. The supernatant was collected, and the protein concentration was determined using the bicinchoninic acid (BCA) method with the Pierce^TM^ BCA Protein Assay Kit (Thermo Fisher, United States). Equal amounts of extracted proteins were loaded and subjected to electrophoresis on 8% SDS-polyacrylamide gels (Beyotime, China) and then transferred to polyvinylidene difluoride (PVDF) membranes (Millipore, United States). Blocking buffer with 5% defatted milk was used to block the membranes for 1 h at room temperature, and membranes were incubated afterward with the following antibodies overnight at 4°C: rabbit anti-LRRK2 (1:10,000, Abcam, United States), rabbit anti-p38 (1:1000, Abcam, United States), rabbit anti-p-p38 (phospho-T180+Y182, 1:400, Abcam, United States), rabbit anti-Drosha (1:1000, Abcam, United States), and chicken anti-albumin (1:1000, Abcam, United States). Rabbit anti-GAPDH (1:10,000, Sigma, United States) was used as an internal loading control. The membranes were then incubated with horseradish peroxidase-conjugated secondary antibodies, including goat anti-rabbit IgG-HRP (Invitrogen, United States) and goat anti-chicken IgG-HRP (Invitrogen, United States), for 2 h at 4°C. Immunoblots were finally probed with the Immobilon^TM^ Western Chemiluminescent HRP Substrate (Millipore, United States) and visualized with an imaging system (Bio-Rad, United States). All data were analyzed using ImageJ software (National Institutes of Health, United States).

### Immunofluorescence Staining

Double immunofluorescence staining was conducted as described previously (Wang et al., 2016). After three washes with 1% Triton in phosphate-buffered saline (PBS) to rupture the cell membranes, frozen brain coronal sections (15 μm) were blocked with 10% goat serum for at least 1 h at room temperature and incubated at 4°C overnight with the following primary antibodies: rabbit anti-LRRK2 (1:50, Abcam, United States), rabbit anti-p38 (1:200, Abcam, United States), rabbit anti-p-p38 (phospho-T180+Y182, 1:80, Abcam, United States), rabbit anti-Drosha (1:1000, Abcam, United States), mouse anti-NeuN (1:200, Millipore, United States), mouse anti-CD11b (1:200, Bio-Rad, United States), and mouse anti-GFAP (1:200, Bio-Rad, United States). The sections were then incubated with secondary antibodies, including Alexa Fluor 488 donkey anti-rabbit IgG antibody (Invitrogen, United States) and Alexa Fluor 555 donkey anti-mouse IgG antibody (Invitrogen, United States), for 1 h at room temperature at a dilution of 1:1000. Finally, slides were counterstained with 4’,6-diamidino-2-phenylindole dihydrochloride (DAPI) for 10 min and were observed with a laser confocal microscope Leica DMi8 (Leica Microsystems, Germany), and images were obtained using LAS X software.

For each animal, three sets of three sections between -2.12 and -4.80 mm from bregma were randomly selected for immunostaining and quantification. The region of interest was defined and delineated under a 10× objective on each section as the LRRK2-, p-p38- or Drosha-positive cells in the contusion margin along the cortex. Using a 20× objective, six randomly selected, nonoverlapping adjacent fields with an area of 690 μm in width and 520 μm in height were examined that surrounded the edge of the contusion cortex. LRRK2-, p-p38-, and Drosha-positive cells and the total number of neurons/microglia/astrocytes were counted manually and expressed as the mean numbers per field of view. The results are presented as percentages of positive cells. All the processes, including sectioning, field selection, and cell counting, were performed by an investigator who was blinded to the treatments among groups.

### Neurological Score

Neurological deficiency was assessed by a blinded investigator 72 h after TBI with the modified Garcia score as previously reported (Garcia et al., 1995; Yang et al., 2016; Feng et al., 2017). Briefly, the following seven parameters were included: spontaneous activity, body proprioception, response to vibrissae touch, symmetry of limb movement, lateral turning, forelimb walking, and climbing ability. Each subtest is scored from 0 to 3, with a composite maximum score of 21 (no neurological deficits).

### Brain Water Content

To study the presence of edema in the injured hemisphere, brain water content was measured using the wet/dry method as described previously (Wu et al., 2016). After surgery and separation of the rat brain tissues, the brains were divided into ipsilateral and contra-lateral frontal hemispheres and quickly weighed to obtain the wet weights. The samples were then placed in a 100°C oven for 72 h to obtain the dry weights. The percentage of brain water content (%) was calculated as [(wet weight-dry weight)/(wet weight)] × 100%.

### TUNEL and FJB Staining

Apoptosis was measured using TUNEL staining according to the manufacturer’s protocol (Abcam, United States). Briefly, brain tissues were fixed with fresh 4% paraformaldehyde at room temperature for 15 min. After being washed with 0.01 M PBS two times, the sections were incubated with 20 μg/ml proteinase K for 5 min at room temperature. Then, the sections were covered with 50 μl of the DNA labeling solution and placed in a dark humidified 37°C incubator for 1 h. After being washed with PBS, slides were immersed in 100 μl of antibody solution in the dark for 30 min at room temperature. Then, sections were washed with ddH_2_O and incubated for 5 min at room temperature. After coverslipping with an anti-fading mounting medium containing DAPI, the fluorescence intensities of the brain sections were assessed immediately with a laser confocal microscope (Leica DMi8, Germany). Three randomly selected sections between -2.12 and -4.80 mm from bregma from each animal were used for quantification. Six nonoverlapping fields surrounding the edge of the contusion cortex were randomly selected and examined at 400× magnification. The results are presented as the apoptosis index, which was quantified as the ratio of (TUNEL-positive cells)/(total cells) × 100%.

Fluoro-Jade B staining served as a marker of neuronal injury and was conducted per the manufacturer’s instructions (Millipore, United States). Briefly, after incubation with 1% sodium hydroxide in 80% alcohol for 5 min and 70% alcohol for 2 min, the slides were transferred to a solution containing 0.06% potassium permanganate for 10 min. The slides were then immersed in 0.0004% Fluoro-Jade dye staining solution (0.1% acetic acid) for 20 min followed by rinsing in deionized water. After being washed and dried in an oven (50°C) for 5–8 min, the sections were cleared by immersion in xylene for at least 1 min before coverslipping with distyrene plasticizer xylene (DPX), a non-aqueous non-fluorescent plastic mounting media. The total number of FJB-positive cells was expressed as the mean number per field of view. All processes, including sectioning, field selection, and cell counting, were conducted by an investigator who was blinded to the animals’ conditions.

### Statistical Analyses

All data were expressed as the mean ± standard deviation and analyzed using SPSS 18.0 software. Statistical analyses of the time course of real-time PCR and western blot data were performed using one-way analysis of variance (ANOVA), followed by Dunnett’s *post hoc* test for comparisons between each TBI group and the sham group. The immunofluorescence staining data were analyzed using Student’s *t*-test. Statistical comparisons among the remaining data were analyzed using one-way ANOVA followed by Tukey’s *post hoc* test to compare data from multiple groups. ANOVA’s *F*-value together with relative degrees of freedom was presented as *F*_(df1,df2)_. *P* < 0.05 was considered to be a statistically significant difference.

## Results

### Time Course of LRRK2, p38, and Drosha Expression in the Peri-Injury Cortex After TBI

**FIGURE 2:**
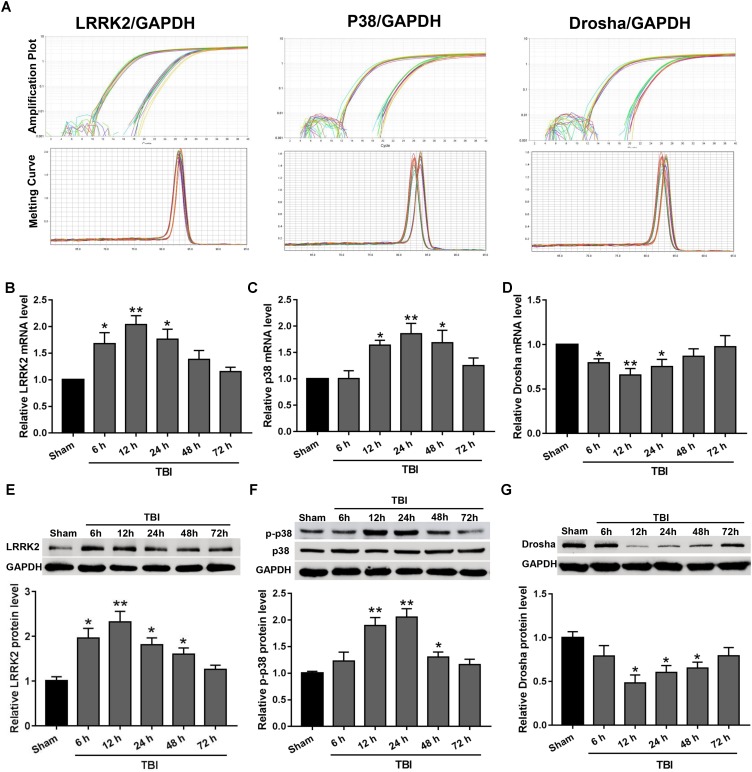
The mRNA and protein expression levels of LRRK2, p38, and Drosha in the peri-injury cortex after TBI. **(A)** Amplification and melting temperature curves for LRRK2, p38, Drosha (right), and GAPDH (left) were obtained to identify the cycle thresholds and confirm the specificity of real-time PCR amplification. The relative mRNA expression levels of **(B)** LRRK2, **(C)** p38, and **(D)** Drosha were evaluated using the ratio of the number of target mRNAs to the GAPDH mRNA. Western blot was performed to determine the protein levels of endogenous **(E)** LRRK2, **(F)** p38, and **(G)** Drosha in the sham and TBI groups at 6, 12, 24, 48, and 72 h. The relative densities of each protein were normalized to the sham group. The results show that the expression levels of LRRK2 and p-p38 were increased at both the mRNA and protein levels after TBI, whereas Drosha expression exhibited the opposite trend. Statistical analyses were performed using one-way analysis of variance (ANOVA) followed by Dunnett’s *post hoc* test. *N* = 6 for each group per time point. Data are expressed as the mean ± SD. ^∗^*P* < 0.05, ^∗∗^*P* < 0.01 vs. sham.

Real-time PCR and western blot analyses were performed to assess the expression levels of endogenous LRRK2, p38, and Drosha at 6, 12, 24, 48, and 72 h following TBI. The amplification plots and melting temperature curves showed the cycle thresholds for these genes and indicated that only one product per gene was generated (**Figure 2A**). The real-time PCR results showed that the mRNA level of LRRK2 was increased beginning at 6 h after TBI and reached its peak at 12 h. Following this peak, the level of LRRK2 mRNA declined gradually and was close to baseline by 48 h (F_(5,30)_ = 6.75, **Figure 2B**). Similar to LRRK2, the level of p38 mRNA increased and peaked at 24 h (*F*_(5,30)_ = 5.59, **Figure 2C**). Conversely, Drosha mRNA declined and reached its minimum at 12 h; the mRNA level of Drosha returned to baseline at 48 h after TBI (*F*_(5,30)_ = 3.59, **Figure 2D**). The results of western blot analysis showed a trend that was consistent with the real-time PCR results (*F*_(5,30)_ = 8.33 for LRRK2, *F*_(5,30)_ = 11.61 for p-p38, *F*_(5,30)_ = 4.18 for Drosha, **Figures 2E–G**).

### Expression of LRRK2, p-p38, and Drosha in Peri-Injury Cortical Cells After TBI

**FIGURE 3:**
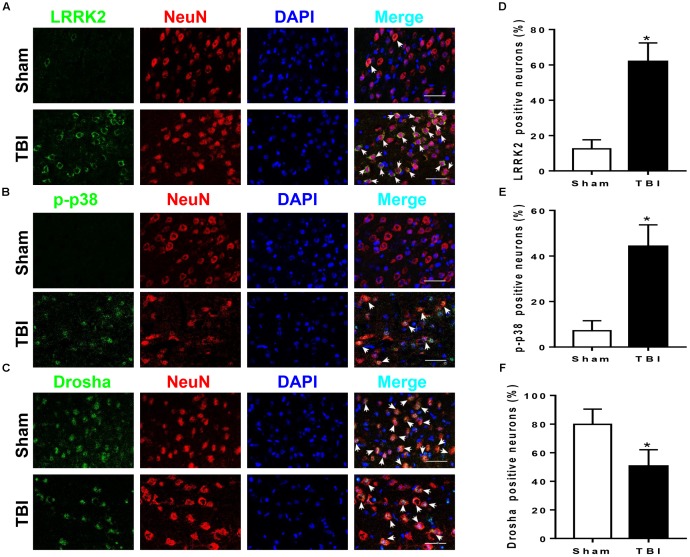
LRRK2, p38, and Drosha expression in neurons of the peri-injury cortex after TBI. Representative double-immunofluorescence staining images of LRRK2 **(A)**, p38 **(B)**, and Drosha **(C)** (green) with NeuN (red)-marked neurons to show expression profiles in the sham and 12-h TBI groups. The nuclei were fluorescently labeled with DAPI (blue). The arrows indicate the colocalization of LRRK2/p-p38/Drosha with neurons. Scale bar = 50 μm. **(D–F)** The percentage of LRRK2/p-p38/Drosha-positive neurons is shown. Statistical analyses were performed using Student’s *t*-test; data are expressed as the mean ± SD, *n* = 6 for each group; ^∗^*P* < 0.05 vs. sham.

LRRK2, p-p38, and Drosha expression were further assessed by immunofluorescence staining with the neuronal marker NeuN, microglial marker CD11b, or astrocytic marker GFAP at 12 h after TBI. Consistent with the western blot results, immunofluorescence analyses revealed that the number of LRRK2 and p-p38-positive neurons in the TBI (12 h) group was remarkably increased, whereas the number of Drosha-positive neurons was reduced compared with that in the sham group (*P* < 0.05, **Figure 3**). In addition, LRRK2, p-p38, and Drosha were also expressed in microglia, and a significant increase in the expression of LRRK2 and p-p38, but not Drosha, was observed at 12 h after TBI (**Supplementary Figure S1**). When double immunostaining with GFAP was performed, only LRRK2 was expressed in astrocytes, and its expression in the TBI (12 h) group was not significantly altered compared to that in the sham group (**Supplementary Figure S2**).

### Effects of LRRK2 Intervention on Brain Edema, BBB Integrity, and Neurological Score After TBI

**FIGURE 4:**
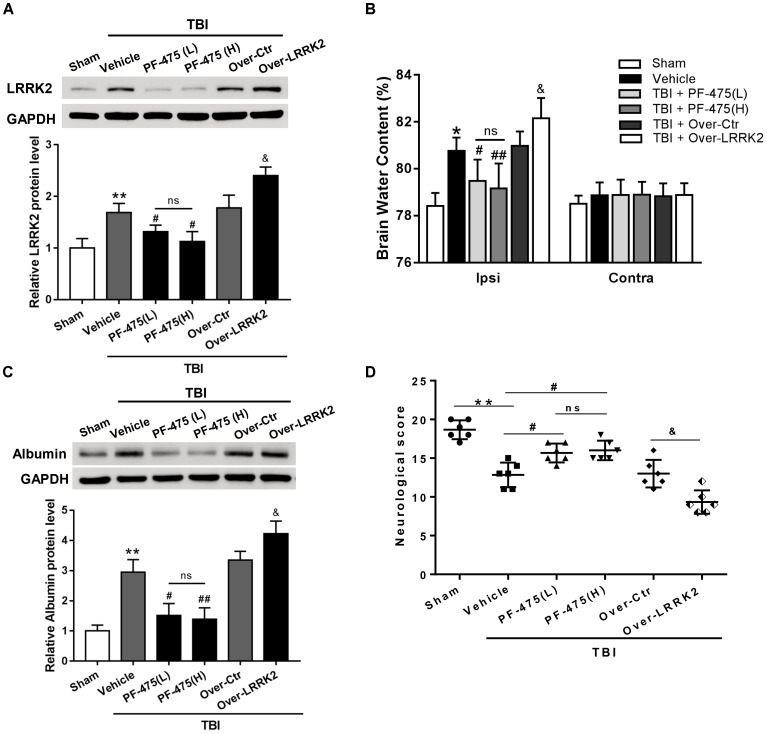
Effects of LRRK2 intervention on neurological score, brain edema, and BBB integrity after TBI. **(A)** Western blot analysis was conducted to assess the efficiencies of LRRK2 intervention in the pericontusional cortex at 12 h after TBI. **(B)** The brain water content of the bilateral hemispheres of the different groups was measured using the wet–dry method. **(C)** Western blots showing the levels of albumin extravasation in the pericontusional cortex at 12 h after TBI. **(D)** Representative neurological behavior scores from the modified Garcia test in the experimental groups at 72 h after TBI are shown. Statistical analyses were performed using one-way ANOVA followed by Tukey’s *post hoc* test. Data are presented as the mean ± SD, *n* = 6 animals per group. ^∗^*P* < 0.05, ^∗∗^*P* < 0.01 vs. sham; ^#^*P* < 0.05, ^##^*P* < 0.01 vs. the Vehicle group; ^&^*P* < 0.05 vs. the Over-Ctr group. Ipsi indicates the ipsilateral injured hemispheres; Contra, contra-lateral uninjured hemispheres.

To verify whether upregulation of LRRK2 was involved in TBI-induced brain injury, we treated rats with different concentrations of LRRK2 inhibitor PF-475 via intraperitoneal injection at 1 h after TBI or with pDNA by ICV for LRRK2 at 24 h before TBI modeling. The results demonstrated that LRRK2 inhibitor PF-475 could markedly decrease the LRRK2 protein level at 12 h after TBI (*F*_(5,30)_ = 31.24, *P* < 0.05, **Figure 4A**). However, there was no significant difference between the low-dose PF-475 (3 mg/kg) and high-dose groups (5 mg/kg) (*P* > 0.05). Conversely, the LRRK2 pDNA pretreatment significantly upregulated LRRK2 expression at 12 h after TBI (*P* < 0.05).

Additionally, we found that brain edema in the injured hemispheres was significantly reduced by the PF-475 treatment and increased by LRRK2 overexpression at 12 h after TBI (*F*_(5,30)_ = 18.49, *P* < 0.05, **Figure 4B**); however, the brain water content did not change significantly in the contra-lateral hemispheres (*F*_(5,30)_ = 0.46, *P* > 0.05). Additionally, we used albumin extravasation to evaluate BBB integrity after TBI. The results showed that albumin extravasation in the injured hemispheres was significantly decreased by LRRK2 inhibition and aggravated by LRRK2 overexpression (*F*_(5,30)_ = 12.31, *P* < 0.05, **Figure 4C**). Consistently, administration of PF-475 significantly improved neurobehavioral deficits, specifically in “symmetry of limb movement” and “lateral turning” evaluation at 72 h after TBI, whereas LRRK2 overexpression exhibited the opposite effect (*F*_(5,30)_ = 29.98, *P* < 0.05, **Figure 4D** and **Supplementary Figure S3**).

### Effects of LRRK2 Intervention on Brain Cell Death and Neuronal Degeneration After TBI

**FIGURE 5:**
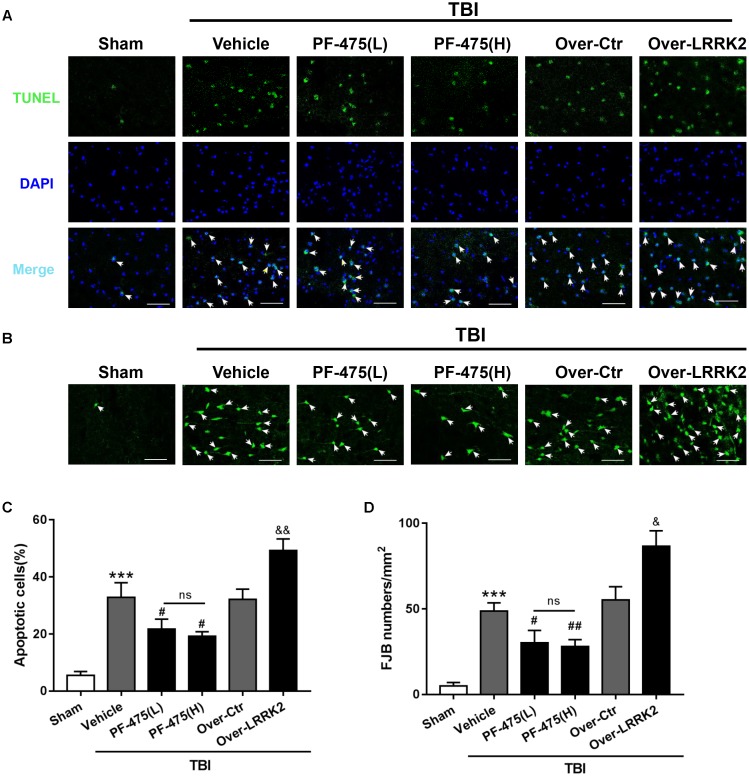
Effects of LRRK2 intervention on brain cell death and neuronal degeneration after TBI. **(A)** Representative photomicrographs of TUNEL staining in the experimental groups are shown. Sections were labeled with TUNEL (green) to assess apoptotic brain cells; sections were counterstained with DAPI (blue) to detect the nuclei. The arrows indicate the TUNEL-positive cells. Scale bar = 50 μm. **(B)** Representative photomicrographs of FJB staining in the experimental groups are shown. The arrows indicate the dead neurons. Scale bar = 50 μm. **(C)** The percentage of TUNEL-positive cells in the brain is shown (apoptotic cells)/(total cells) × 100%. **(D)** The neuronal degeneration index is presented as the number of FJB-positive cells per visual field. Statistical analyses were performed using one-way ANOVA followed by Tukey’s *post hoc* test. Data are expressed as the mean ± SD, *n* = 6 animals per group. ^∗∗∗^*P* < 0.001 vs. sham; ^#^*P* < 0.05, ^##^*P* < 0.01 vs. the Vehicle group; ^&^*P* < 0.05, ^&&^*P* < 0.01 vs. the Over-Ctr group.

**FIGURE 6:**
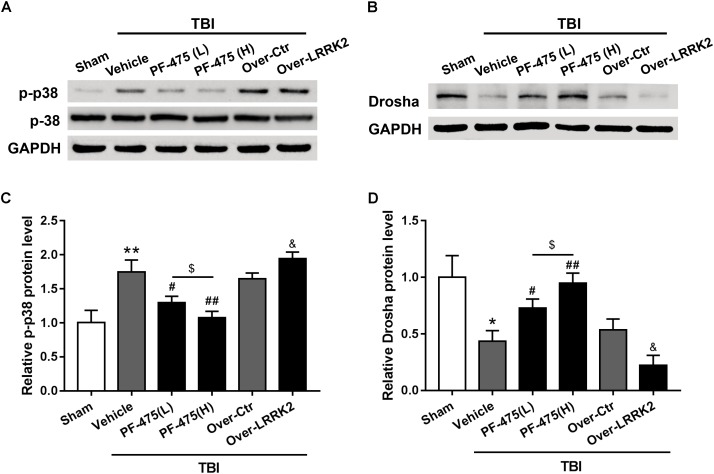
Effects of LRRK2 intervention on the p-p38 and Drosha protein levels after TBI. Western blot analysis was conducted to assess the **(A)** p-p38 and **(B)** Drosha protein levels after LRRK2 intervention. **(C,D)** The densities of the protein bands in the representative images were analyzed and normalized to GAPDH. The PF-475 treatment reversed the TBI-induced increase in p-p38 and decrease in Drosha expression, whereas LRRK2 overexpression augmented the TBI-induced changes in p-p38 and Drosha expression. Statistical analyses were performed using one-way ANOVA followed by Tukey’s *post hoc* test. Data are presented as the mean ± SD, *n* = 6 animals per group. ^∗^*P* < 0.05, ^∗∗^*P* < 0.01 vs. sham; ^#^*P* < 0.05, ^##^*P* < 0.01 vs. the Vehicle group; ^&^*P* < 0.05 vs. the Over-Ctr group; ^$^*P* < 0.05 vs. the indicated group.

To assess the role of LRRK2 in TBI-induced neuronal apoptosis, we performed TUNEL and FJB staining to evaluate cell death and neuronal degeneration, respectively, in the peri-injury cortex at 12 h after TBI. As shown in **Figure 6**, treatment with PF-475 to inhibit LRRK2 grossly reduced the number of TUNEL-positive cells, although the treatment did not eliminate apoptotic cells completely (*F*_(5,30)_ = 55.14, *P* < 0.05, **Figures 5A,C**). However, LRRK2 overexpression further augmented the number of apoptotic cells at 12 h after TBI (*P* < 0.05). In accordance with the TUNEL results, FJB staining showed that TBI-induced neuronal degeneration was significantly inhibited by the PF-475 treatment and enhanced by LRRK2 overexpression (*F*_(5,30)_ = 58.24, *P* < 0.05, **Figures 5B,D**).

### Effects of LRRK2 Intervention on Expression of p-p38 and Drosha After TBI

To determine whether LRRK2 played a role in regulating the p38 MAPK pathway and its downstream molecule Drosha, western blot analyses of the peri-injury cortex were performed, and changes in the protein levels of p-p38 and Drosha were quantified following LRRK2 inhibition or overexpression at 12 h after TBI or sham surgery. Our results demonstrated that LRRK2 inhibitor PF-475 decreased p-p38 expression in a dose-dependent manner (*F*_(5,30)_ = 27.13, *P* < 0.05, **Figures 6A,C**), whereas LRRK2 overexpression significantly enhanced the p-p38 level. Conversely, the expression of Drosha, which was upregulated with the PF-475 treatment (*F*_(5,30)_ = 21.58, *P* < 0.05, **Figures 6B,D**) and greatly downregulated in the LRRK2 overexpression group (*P* < 0.05), showed an opposite trend to that of p-p38.

### Simultaneous Overexpression of Drosha Abolishes the Augmenting Effects of LRRK2 Overexpression on Brain Edema, BBB Integrity, and Neurologic Impairment After TBI

**FIGURE 7:**
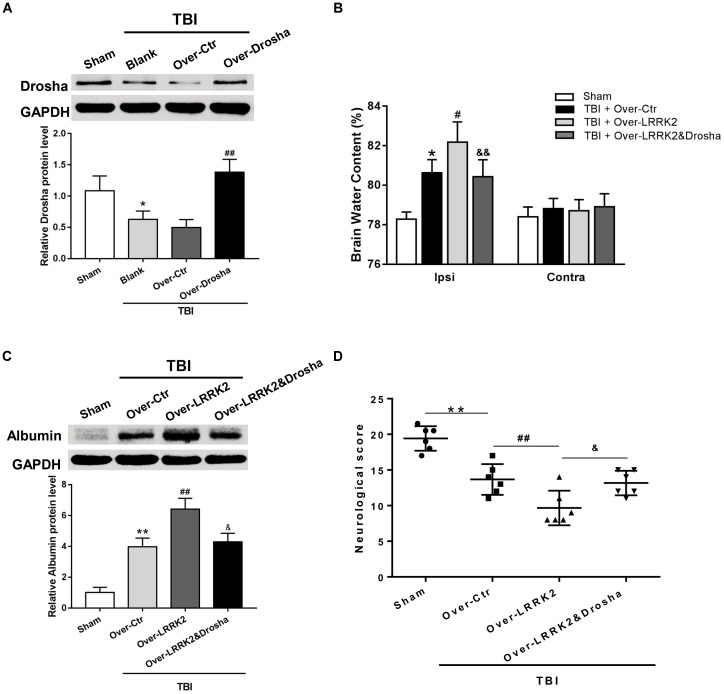
Simultaneous overexpression of Drosha abolishes the neurotoxic effects of LRRK2 overexpression on neurological impairment, brain edema, and BBB integrity after TBI. **(A)** Western blot analysis was conducted to assess the Drosha overexpression efficiency and the effect of LRRK2 expression in the pericontusional cortex at 12 h after TBI. The results showed that Drosha overexpression pretreatment effectively raised the Drosha protein level but did not change the expression of LRRK2. Meanwhile, the neurotoxic effects of LRRK2 overexpression on **(B)** brain edema, **(C)** albumin leakage, and **(D)** neurological score were reversed by simultaneous Drosha overexpression. Statistical analyses were performed using one-way ANOVA followed by Tukey’s *post hoc* test. Data are presented as the mean ± SD, *n* = 6 animals per group. ^∗^*P* < 0.05, ^∗∗^*P* < 0.01 vs. sham, ^#^*P* < 0.05, ^##^*P* < 0.01 vs. the Over-Ctr group; ^&^*P* < 0.05, ^&&^*P* < 0.01 vs. the Over-LRRK2 group.

To further verify the role of Drosha in LRRK2-induced brain injury, we simultaneously administered the pDNAs of Drosha and LRRK2. The overexpression efficiency of Drosha was demonstrated by western blot. Pretreatment with Drosha pDNA significantly increased Drosha expression (*F*_(3,20)_ = 17.26, *P* < 0.05, **Figure 7A**) but had no effect on the expression of LRRK2 in the ipsilateral hemisphere at 12 h after TBI (*F*_(3,20)_ = 12.65, *P* > 0.05, **Figure 7A**). Additionally, compared with the LRRK2 overexpression treatment alone, the Drosha overexpression pretreatment sufficiently abolished the neurotoxic effects of LRRK2 on brain edema (*F*_(3,20)_ = 25.97, *P* < 0.05, **Figure 7B**) and BBB disruption at 12 h after TBI (*F*_(3,20)_ = 21.95, *P* < 0.05, **Figure 7C**), as well as neurological deficits, specifically in “symmetry of limb movement” and “lateral turning” evaluation, at 72 h after TBI (*F*_(3,20)_ = 23.81, *P* < 0.05, **Figure 7D** and **Supplementary Figure S4**).

### Simultaneous Overexpression of Drosha Reverses the Augmenting Effects of LRRK2 Overexpression on Brain Cell Death and Neuronal Degeneration After TBI

**FIGURE 8:**
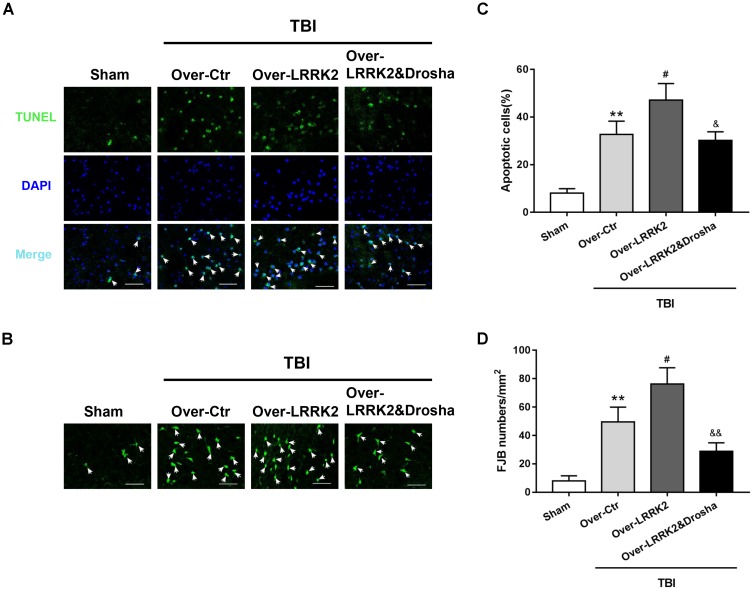
Simultaneous overexpression of Drosha abolishes the neurotoxic effects of LRRK2 overexpression on brain cell death and neuronal degeneration after TBI. Representative photomicrographs of **(A)** TUNEL and **(B)** FJB staining of the pericontusional cortex in the experimental groups are shown. The arrows indicate the TUNEL-positive or FJB-positive cells. Scale bar = 50 μm. **(C)** The apoptosis index is expressed as the ratio of (apoptotic cells)/(total cells) × 100%. **(D)** The neuronal degeneration index is presented as the number of FJB-positive cells per visual field. Statistical analyses were performed using one-way ANOVA followed by Tukey’s *post hoc* test. Data are presented as the mean ± SD, *n* = 6 animals per group. ^∗∗^*P* < 0.01 vs. sham; ^#^*P* < 0.05 vs. the Over-Ctr group; ^&^*P* < 0.05, ^&&^*P* < 0.01 vs. the Over-LRRK2 group.

Terminal deoxynucleotidyl transferase-mediated dUTP nick-end labeling staining analysis showed that simultaneous Drosha overexpression completely reversed the neurotoxic effects of LRRK2 on brain cell death compared with LRRK2 overexpression treatment alone (*F*_(3,20)_ = 32.98, *P* < 0.05, **Figures 8A,C**). Consistently, Drosha overexpression ameliorated neuronal degeneration compared with the LRRK2 overexpression group (*F*_(3,20)_ = 34.91, *P* < 0.05, **Figures 8B,D**).

## Discussion

In this study, we investigated the role of LRRK2 in TBI-induced secondary brain injury and elucidated the potential underlying mechanisms in a rat model of TBI. Our findings showed that following TBI, the expression level of endogenous LRRK2 and phosphorylation level of p38 was increased, whereas the expression level of Drosha was reduced. Inhibiting LRRK2 with PF-475 reduced neuronal apoptosis, brain edema, BBB permeability, and alleviated neurological deficits, which were concomitant with decreased p38 phosphorylation and increased Drosha expression. Conversely, LRRK2 overexpression exacerbated TBI-induced brain injury and aggravated the increase in p-p38 activity and reduction in the Drosha protein level. Furthermore, combined overexpression of Drosha eliminated the toxic effects of LRRK2 overexpression on neurological injury. Taken together, these observations suggest that LRRK2 contributes to secondary brain injury after TBI, at least in part, via a p38/Drosha signaling pathway.

To date, studies related to LRRK2 in central nervous system (CNS) are mainly focused on PD. Cumulative evidence suggests that LRRK2 plays key roles in axonal extension, autophagy, oxidative stress, and survival in neurons (Tsika and Moore, 2012; Bae and Lee, 2015; Cookson, 2015). Overexpression of wild-type LRRK2 enhances mitochondrial dynamin-related protein 1-mediated mitochondrial fragmentation, mitochondrial dysfunction, and neuronal toxicity in primary neuronal cultures (Wang et al., 2012). Conversely, LRRK2 knockout attenuates the neuropathology that is induced by α-synuclein overexpression in the mouse brain through a delay in neuronal death that results from an improved structure and function of the Golgi complex (Lin et al., 2009). Notably, LRRK2 may contribute to neuronal apoptosis following cerebral ischemia by modulating the phosphorylation of Tau, a microtubule-associated protein that is predominantly expressed in the CNS and that regulates neurite outgrowth and axonal transport (Kim and Vemuganti, 2017). Similar to the neurotoxicity effects of LRRK2 in experimental PD, our study showed that both the mRNA and protein levels of LRRK2 were increased in the injured brain region and peaked at 12 h after TBI, while inhibiting LRRK2 expression ameliorated TBI-induced neuronal apoptosis, brain edema, BBB permeability, and neurological impairment. Conversely, LRRK2 overexpression aggravated these pathological processes. These findings were consistent with the role of LRRK2 in the experimental cerebral ischemia model and indicate that upregulation of LRRK2 contributes to secondary brain injury after TBI.

However, to date, little is known about the exact molecular mechanisms involved in LRRK2-mediated neurotoxicity in TBI. Specifically, due to the sequence homology of its kinase domain with stress-activated mixed lineage kinases, LRRK2 is implicated in the upstream functioning in the MAPK signaling cascades (Gloeckner et al., 2009; Hsu et al., 2010a), which play central roles in neuronal survival and death after brain injury (Raghupathi et al., 2003). MAPKs are serine/threonine kinases that include extracellular signal-regulated kinases (ERKs), p38 MAPK, and c-Jun N-terminal kinases (JNKs). Among them, p38 MAPK is a pro-apoptotic signaling pathway (Yang et al., 2017). Activation of p38 leads to the production of cleaved caspase-3 and a reduction in bcl-2, thus resulting in apoptotic neuronal death (Raghupathi et al., 2003). Previous studies have shown significant activation of p38 in the brain after TBI (Tang et al., 2012; Wei et al., 2014; Li et al., 2017; Yang et al., 2017). Blocking p38 activation attenuates matrix metalloprotein-9 (MMP-9) expression and thus mitigates brain edema following TBI (Tang et al., 2012). In agreement with these results, we found that TBI induced a rapid increase in p-p38 activity, which was reversed by inhibiting LRRK2 and further enhanced by overexpressing LRRK2. These data indicate that p38 pathway activation may be involved in LRRK2-induced brain injury after TBI.

As a nuclear RNase III enzyme, Drosha controls the initial step of miRNA biogenesis (Aguado et al., 2017; Mechtler et al., 2017). miRNAs have the ability to orchestrate the maintenance of adult neural cell traits, promote cellular homeostasis, and dampen endogenous and exogenous stress responses (Yokota, 2009). Therefore, the stability of Drosha in the brain is closely related to neuronal survival. A previous study showed that disrupting the stability of Drosha using DNA-/RNA-binding proteins could induce cytotoxicity in neuronal cultures (Kim et al., 2015). Here, we observed that the expression of Drosha was significantly downregulated after TBI, suggesting that a Drosha deficiency might also play a role in neurological damage following TBI. In contrast to our study, one report indicated that the protein level of Drosha was not altered after transient middle cerebral artery occlusion in a transient focal ischemia model (Dharap et al., 2009). This discrepancy might be due to differences in animal models, time points, measured areas, or experimental conditions. Remarkably, under stress conditions, activated p38 can inhibit the function of Drosha and promote its nuclear export and degradation by calpain, which eventually sensitizes cells to stress and increases apoptotic cell death (Yang et al., 2015). Hence, we speculate that the toxic effects of LRRK2 on brain injury may be related to p38-mediated Drosha inhibition. Unsurprisingly, our observations showed that Drosha expression following TBI was further inhibited by LRRK2 overexpression and was markedly enhanced by LRRK2 inhibition, which was completely opposite to the trend of p-p38 activity. Meanwhile, LRRK2 expression was not affected by the overexpression of Drosha, which suggests that Drosha is a downstream effector of LRRK2. In addition, combining overexpression of Drosha with LRRK2 abolished the neurotoxicity of LRRK2 after TBI. Taken together, these findings support our hypothesis and suggest that LRRK2 elevation may inhibit Drosha expression by activating the p38 signaling pathway, thus aggravating secondary brain injury after TBI in rats.

Multiple studies have indicated that LRRK2 also participates in neuroinflammation by activating microglia (Lee et al., 2017). After inflammation induced by lipopolysaccharide, a robust induction of LRRK2 protein expression was observed in microglial cells (Gillardon et al., 2012; Moehle et al., 2012). LRRK2 phosphorylated p53 in microglia and then increased the secretion of the pro-inflammatory cytokine TNFα (Ho et al., 2017). In addition, knockdown of LRRK2 expression or pharmacological inhibition in microglia has been shown to alleviate pro-inflammatory signaling, including reduced levels of iNOS induction, p-p38, and transcriptional activity of NF-κB (Kim et al., 2012; Moehle et al., 2012). In this study, we observed that LRRK2 co-localized with microglia and the number of LRRK2-positive microglia was significantly increased after TBI. These findings imply that LRRK2 may play a role in neuroinflammation, which is an established pathological process that contributes to secondary brain injury after TBI (Casili et al., 2018). However, the exact functions of LRRK2 in microglia and in neuroinflammation following TBI remain unclear. Therefore, the above issues need to be clarified in our future study.

There are several limitations worth mentioning in the current study. First, in addition to the p38 pathway, LRRK2 exerts multiple neurotoxic effects via different signaling pathways in the nervous system. For example, the other two classic MAPK signaling cascades, JNKs and ERKs, which were not examined in this study, are also involved in the neurotoxicity of LRRK2 (Hsu et al., 2010b; Verma et al., 2014). Second, altered synaptic plasticity is another neurobiological substrate of neuronal loss in a number of neurological diseases including TBI (Zhang et al., 2011). LRRK2 has been shown to promote the synaptic translocation of protein kinase A and increased protein kinase A-mediated phosphorylation of the actin-disassembling enzyme cofilin and the glutamate receptor GluR1, resulting in abnormal synaptogenesis and transmission in neurons (Parisiadou et al., 2014). Hence, we cannot exclude the possibility that the aberrant synaptic machinery also plays a role in the neuronal toxicity of LRRK2 in neurological injury after TBI. Another limitation in our study was that only male rats were utilized. Thus, we were unable to investigate gender differences in LRRK2 expression or gender effects in TBI. Therefore, we should be cautious when interpreting these results.

## Conclusion

**FIGURE 9:**
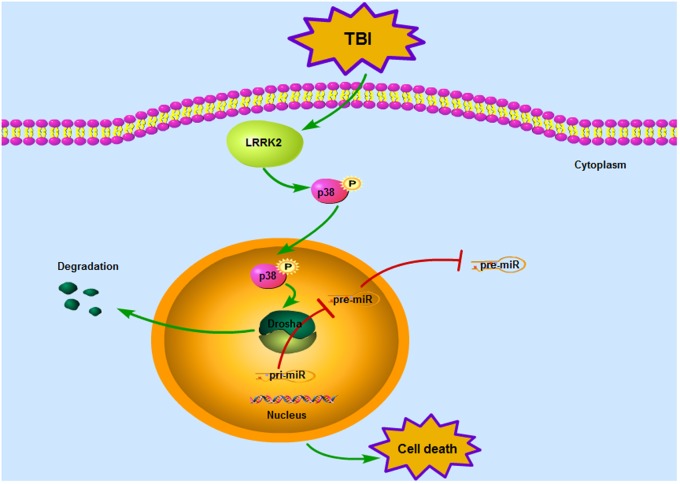
Mode pattern illustrating the possible mechanisms underlying LRRK2-mediated neurotoxicity after TBI. Briefly, TBI induces a rapid increase in the expression of endogenous LRRK2, which can activate the p38 pathway. Phosphorylated p38 then translocates from the cytoplasm to the nucleus and disrupts the stability of Drosha, promoting its nuclear export and degradation. As a nuclear RNase III enzyme, Drosha deficiency in the nucleus can hinder miRNA maturation, which eventually sensitizes cells to stress and results in neurological injury after TBI.

Our observations indicate for the first time that LRRK2 may contribute to secondary brain injury by suppressing the expression of a key enzyme in miRNA biogenesis, Drosha, via the p38 pathway after TBI (**Figure 9**). This study provides new mechanistic information regarding the pathological process of TBI and suggests that targeting LRRK2 will pave the way for a new therapeutic strategy for TBI patients.

## Ethics Statement

All experimental protocols were approved by the Animal Care and Use Committee of Soochow University and were implemented with reference to the ARRIVE guidelines. This article does not contain any studies with human participants performed by any of the authors.

## Author Contributions

RG and GC designed the research. QR, HN, and DL performed the entire experiments. FG and BD analyzed the data. QR and HN wrote the manuscript together.

## Conflict of Interest Statement

The authors declare that the research was conducted in the absence of any commercial or financial relationships that could be construed as a potential conflict of interest.

## Supplementary Material

**FIGURE S1 |** LRRK2, p38, and Drosha expression in microglia of the peri-injury cortex after TBI. Representative double-immunofluorescence staining images of LRRK2 **(A)**, p38 **(B)**, and Drosha **(C)** (green) with microglial marker CD11b (red) to show expression profiles in the sham and 12 h TBI groups. The nuclei were fluorescently labeled with DAPI (blue). The arrows indicate the colocalization of LRRK2/p-p38/Drosha with microglia. Scale bar = 50 μm. **(D–F)** The percentage of LRRK2/p-p38/Drosha-positive microglia is shown. Statistical analyses were performed using Student’s *t*-test; data are expressed as the mean ± SD, *n* = 6 for each group; ^∗^*P* < 0.05 vs. sham.

**FIGURE S2 |** LRRK2, p38, and Drosha expression in astrocytes of the peri-injury cortex after TBI. Representative double-immunofluorescence staining images of LRRK2 **(A)**, p38 **(B)**, and Drosha **(C)** (green) with astrocytic marker GFAP (red)-marked astrocytes to show expression profiles in the sham and 12 h TBI groups. The nuclei were fluorescently labeled with DAPI (blue). The arrows indicate the colocalization of LRRK2/p-p38/Drosha with astrocytes. Scale bar = 50 μm. **(D)** The percentage of LRRK2-positive astrocytes is shown. Statistical analyses were performed using Student’s *t*-test; data are expressed as the mean ± SD, *n* = 6 for each group; ^∗^*P* < 0.05 vs. sham.

**FIGURE S3 |** Effects of LRRK2 intervention on separated behavioral evaluation of neurological deficiency after TBI. Representative seven parameters of the modified Garcia score include the following: **(A)** spontaneous activity, **(B)** body proprioception, **(C)** response to vibrissae touch, **(D)** symmetry of limb movement, **(E)** lateral turning, **(F)** forelimb walking, and **(G)** climbing ability. Statistical analyses were performed using one-way ANOVA followed by Tukey’s *post hoc* test; *n* = 6 rats for each group per time point. Data are expressed as the mean ± SD. ^∗^*P* < 0.05, ^∗∗^*P* < 0.01 vs. sham, ^&^*P* < 0.05 vs. Vehicle, ^#^*P* < 0.05, ^##^*P* < 0.01 vs. Over-Ctr.

**FIGURE S4 |** Effects of simultaneous overexpression of Drosha and LRRK2 on separated behavioral evaluation of neurological deficiency after TBI. Representative seven parameters of the modified Garcia score include the following: **(A)** spontaneous activity, **(B)** body proprioception, **(C)** response to vibrissae touch, **(D)** symmetry of limb movement, **(E)** lateral turning, **(F)** forelimb walking, and **(G)** climbing ability. Statistical analyses were performed using one-way ANOVA followed by Tukey’s *post hoc* test; *n* = 6 rats for each group per time point. Data are expressed as the mean ± SD. ^∗^*P* < 0.05, ^∗∗^*P* < 0.01 vs. sham, ^#^*P* < 0.05, ^##^*P* < 0.01 vs. Over-Ctr.
