# Supplementary figures and images for: LRRK2 Contributes to Secondary Brain Injury Through a p38/Drosha Signaling Pathway After Traumatic Brain Injury in Rats
Source: Front Cell Neurosci. 2018 Mar 1;12:51. doi: 10.3389/fncel.2018.00051 (PMC5837969; doi:10.3389/fncel.2018.00051)

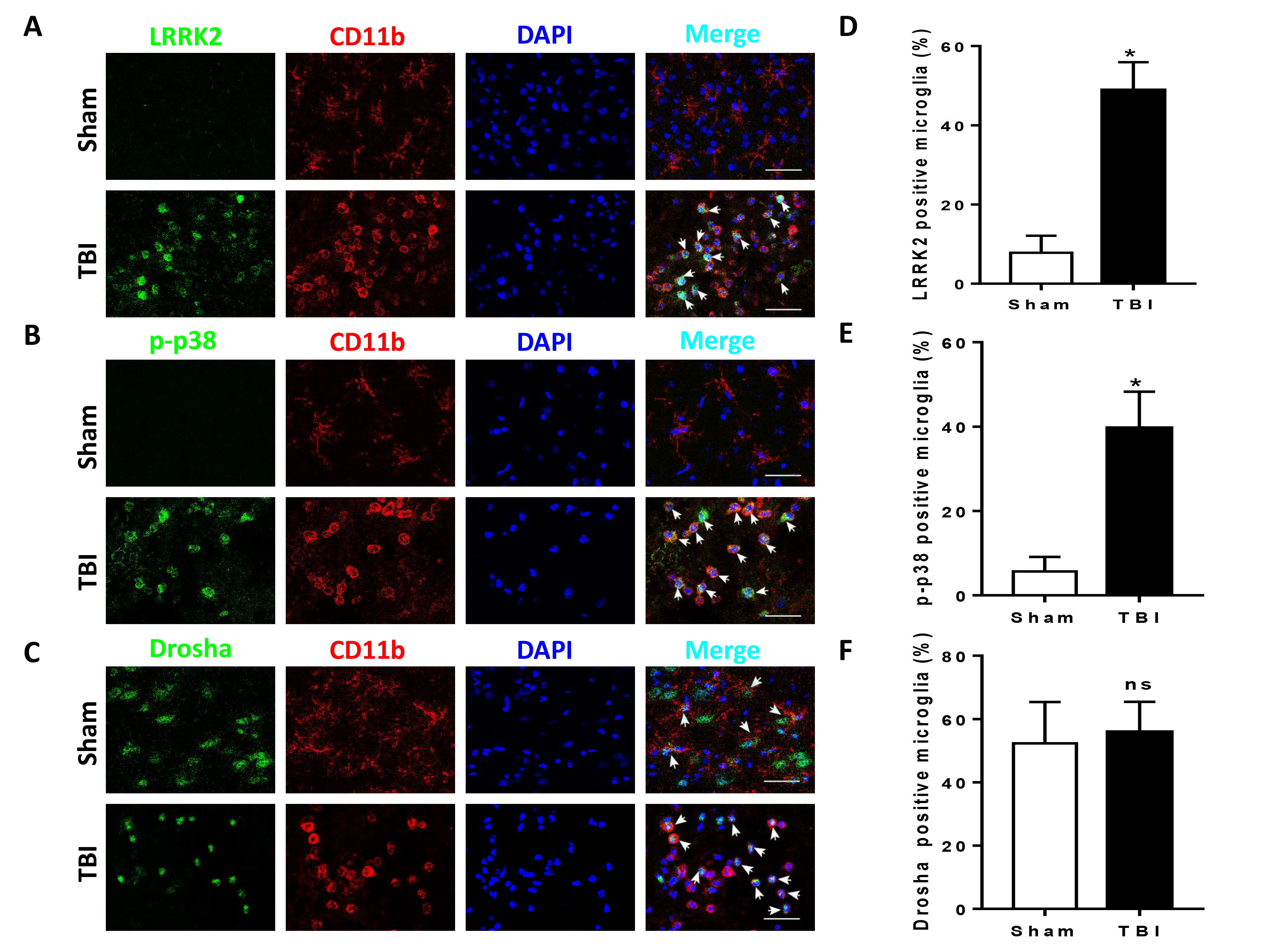

Supplement: Supplementary file 1 [file Image_1.TIF]

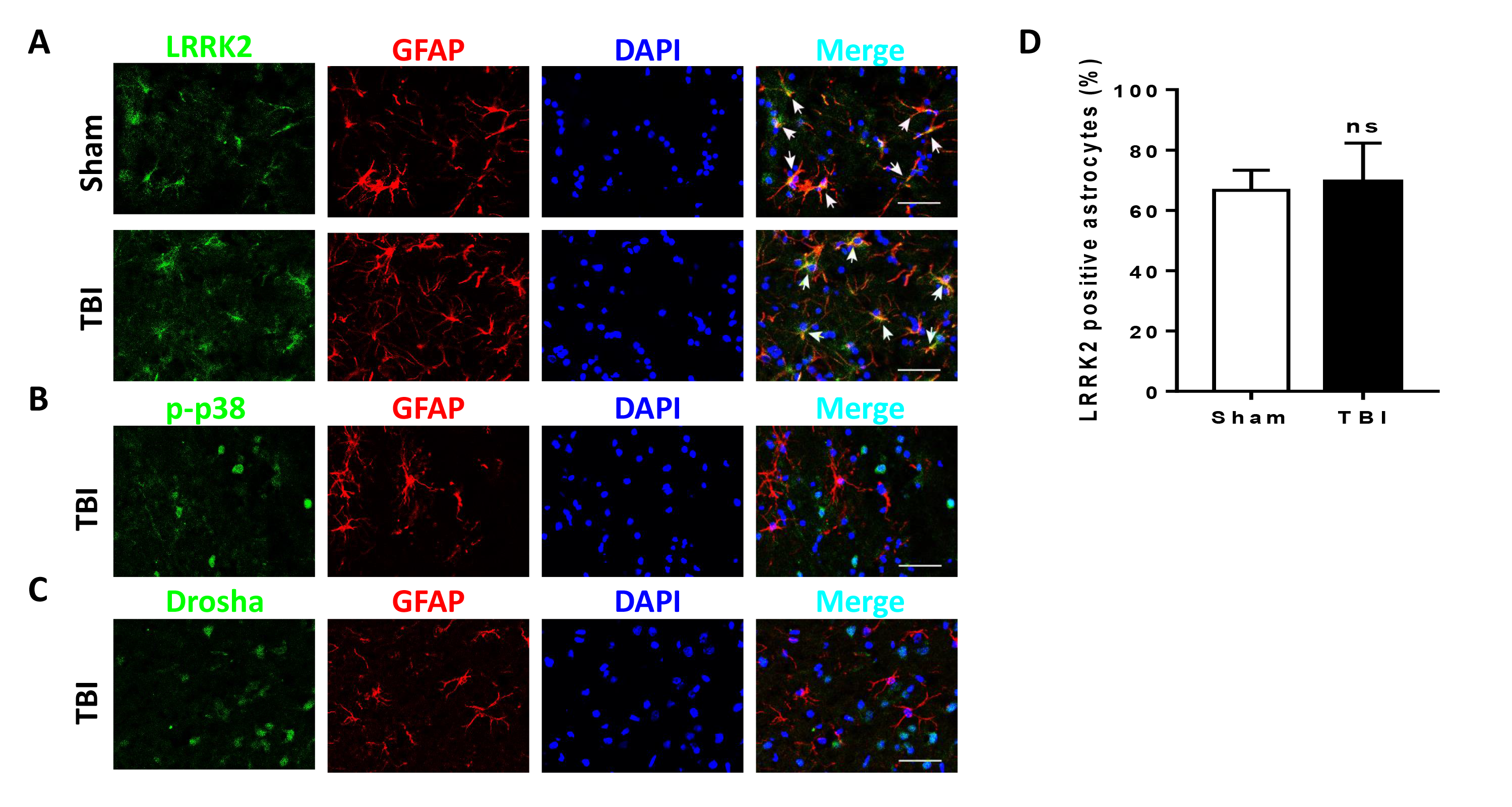

Supplement: Supplementary file 2 [file Image_2.TIF]

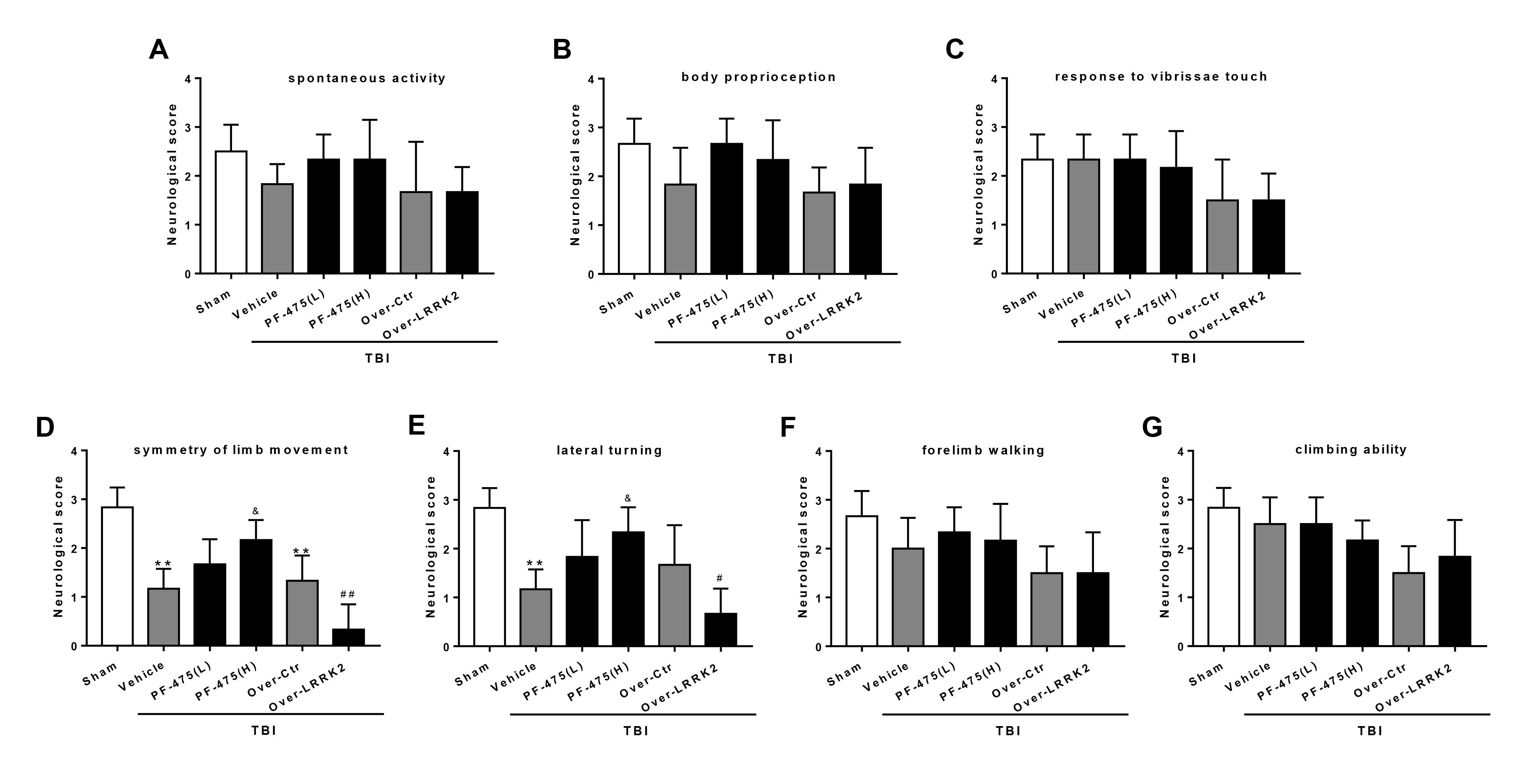

Supplement: Supplementary file 3 [file Image_3.TIF]

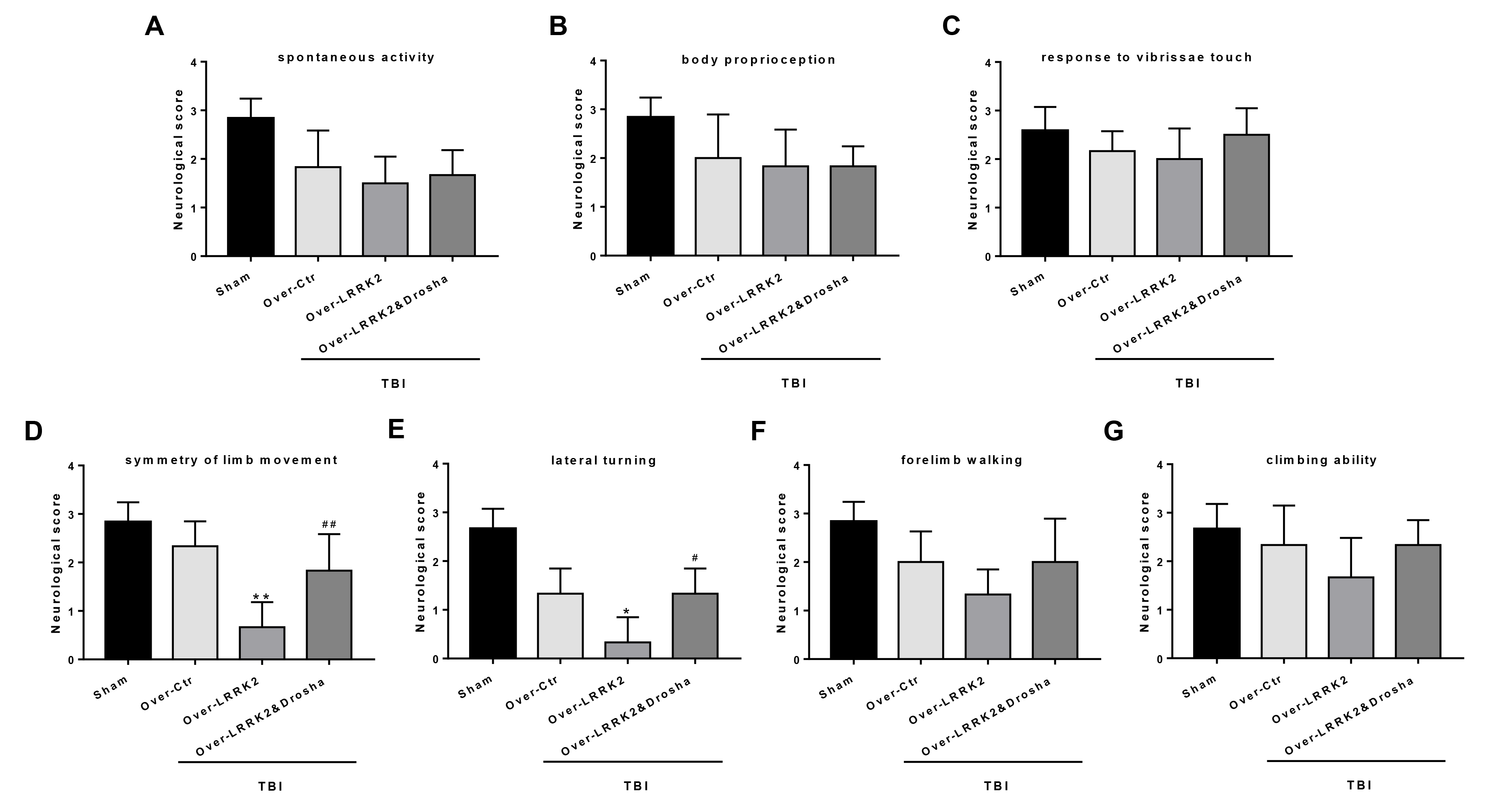

Supplement: Supplementary file 4 [file Image_4.TIF]
